# A Novel Model Based on Deep Convolutional Neural Network Improves Diagnostic Accuracy of Intramucosal Gastric Cancer (With Video)

**DOI:** 10.3389/fonc.2021.622827

**Published:** 2021-04-20

**Authors:** Dehua Tang, Jie Zhou, Lei Wang, Muhan Ni, Min Chen, Shahzeb Hassan, Renquan Luo, Xi Chen, Xinqi He, Lihui Zhang, Xiwei Ding, Honggang Yu, Guifang Xu, Xiaoping Zou

**Affiliations:** ^1^ Department of Gastroenterology, Nanjing Drum Tower Hospital, Affiliated Drum Tower Hospital, Medical School of Nanjing University, Nanjing, China; ^2^ Department of Gastroenterology, Renmin Hospital of Wuhan University, Wuhan, China; ^3^ Key Laboratory of Hubei Province for Digestive System Disease, Renmin Hospital of Wuhan University, Wuhan, China; ^4^ Hubei Provincial Clinical Research Center for Digestive Disease Minimally Invasive Incision, Renmin Hospital of Wuhan University, Wuhan, China; ^5^ Northwestern University Feinberg School of Medicine, Chicago, IL, United States

**Keywords:** artificial intelligence, deep convolutional neural network, depth of invasion, gastric cancer, endoscopic resection

## Abstract

**Background and Aims:**

Prediction of intramucosal gastric cancer (GC) is a big challenge. It is not clear whether artificial intelligence could assist endoscopists in the diagnosis.

**Methods:**

A deep convolutional neural networks (DCNN) model was developed *via* retrospectively collected 3407 endoscopic images from 666 gastric cancer patients from two Endoscopy Centers (training dataset). The DCNN model’s performance was tested with 228 images from 62 independent patients (testing dataset). The endoscopists evaluated the image and video testing dataset with or without the DCNN model’s assistance, respectively. Endoscopists’ diagnostic performance was compared with or without the DCNN model’s assistance and investigated the effects of assistance using correlations and linear regression analyses.

**Results:**

The DCNN model discriminated intramucosal GC from advanced GC with an AUC of 0.942 (95% CI, 0.915–0.970), a sensitivity of 90.5% (95% CI, 84.1%–95.4%), and a specificity of 85.3% (95% CI, 77.1%–90.9%) in the testing dataset. The diagnostic performance of novice endoscopists was comparable to those of expert endoscopists with the DCNN model’s assistance (accuracy: 84.6% *vs.* 85.5%, sensitivity: 85.7% *vs.* 87.4%, specificity: 83.3% *vs.* 83.0%). The mean pairwise kappa value of endoscopists was increased significantly with the DCNN model’s assistance (0.430–0.629 *vs.* 0.660–0.861). The diagnostic duration reduced considerably with the assistance of the DCNN model from 4.35s to 3.01s. The correlation between the perseverance of effort and diagnostic accuracy of endoscopists was diminished using the DCNN model (r: 0.470 *vs.* 0.076).

**Conclusions:**

An AI-assisted system was established and found useful for novice endoscopists to achieve comparable diagnostic performance with experts.

## Introduction

Gastric cancer (GC) patients are mostly diagnosed at an advanced stage and are ineligible for curative resection, making it the third leading cause of cancer deaths worldwide (1). But if GC can be diagnosed and then curatively resected at an early stage, the 5-year survival rate of this malignancy exceeds 95% (2). Various studies have validated that endoscopic submucosal dissection (ESD) can be available to treat early gastric cancer (3–6). According to the Japanese Gastric Cancer Treatment Guidelines 2018, the absolute indications for ESD of early gastric cancer include differentiated intramucosal cancer without ulceration and differentiated intramucosal cancer with ulceration and tumor size of ≤ 3 cm (7). Previous studies demonstrated that the incidence of lymph node metastasis (LNM) of these intramucosal gastric cancer lesions is negligible (8, 9). Therefore, it is of great essence to determine whether there is deep submucosal invasion before gastric ESD. However, it remains a challenge to distinguish intramucosal gastric cancer lesions from submucosal lesions correctly.

In clinical practice, invasion depth of gastric cancer is often determined by assessing the macroscopic features using conventional white-light imaging (C-WLI) endoscopy or evaluating the linings and walls using endoscopic ultrasonography (EUS). However, various studies have demonstrated that the diagnostic performance of macroscopic features using C-WLI and linings and walls with EUS in invasion depth was comparable, with a limited accuracy of only 70–85% (10, 11). More than 15% of gastric cancer lesions have been underestimated or overestimated using both methodologies. Although enhanced imaging technologies like magnifying endoscopy (ME), narrow-band imaging (NBI), and blue laser imaging (BLI) have also been employed in the determination of intramucosal GC, the clinical value of these techniques largely depends on the experience of operators (12, 13). Moreover, the accuracy and concordance of all the methodologies were reported to vary wildly in different studies, even amongst the expertized endoscopists (10, 14, 15). Therefore, it would be very advantageous to develop efficient assistance tools to help endoscopists make robust, reproducible, and accurate diagnoses of intramucosal GC under C-WLI.

With recent technological advances, artificial intelligence (AI) has shown excellent efficacy in analyzing medical images (16). Several preclinical studies reported that AI could be used with high accuracy for detection, localization, and classification of GC (17–19). Three preliminary studies have applied AI to predict the invasion depth of GC with acceptable specificity or sensitivity (18, 20, 21). However, these studies only focused on evaluating AI’s performance in predicting invasion depth instead of verifying AI’s assistance in helping endoscopists make the final diagnosis. The latter is even more important than the former since endoscopists are required to make the final diagnosis due to safety and accountability.

This study aimed to develop an AI-assisted diagnostic model based on the deep convolutional neural networks (DCNN) to detect intramucosal GC from advanced lesions in real-time. We then evaluated the accuracy, concordance, and diagnostic duration of the DCNN model’s assistance in helping endoscopists establish the final diagnosis.

## Methods

### Study Design

This retrospective comparative study was performed at two institutions in China: Endoscopy Center of Nanjing University Medical School Affiliated Drum Tower Hospital (NJDTH) and Endoscopy Center of Renmin Hospital of Wuhan University (RHWU). We first trained our DCNN model to distinguish intramucosal gastric cancer lesions from submucosal lesions. Then, we assessed the performance of DCNN and evaluated the performance of endoscopists before (Test 1) and after referring to the DCNN-processed results (Test 2) with endoscopic images and videos. The study design was reviewed and approved by the Medical Ethics Committee at each institution (NJDTH, IRB no. 2020-026-01; RHWU, WDRY2019-K091). Informed consent was waived given the use of only retrospectively deidentified endoscopic images.

### Data Preparation and Image Quality Control

A total of 870 patients who underwent endoscopic submucosal dissection (ESD) or gastrectomy with histologically proven malignancies (700 patients from NJDTH and 170 patients from RHWU) between Jan 2017 and June 2019 were retrospectively included in this study. After excluding patients with multiple synchronous lesions, gastric stump cancer, and missing data, 3829 endoscopic images from 728 patients were obtained retrospectively from the imaging database of the two hospitals. A total of 194 endoscopic images were excluded from the study due to low quality (e.g., less insufflation of air, halation, defocus, blurs, bubble, sliding, fuzzy, and bleeding). The rest of the 3635 endoscopic images (from 728 patients) were used to develop and validate the AI model (**Table S1**). Moreover, 54 videos with single GC lesions of another 54 patients from NJDTH between Jan 2019 and June 2019, which were independent of 700 patients, were retrospectively collected in this study and used to test the AI’s performance model and endoscopists. All the endoscopic images and videos were recorded with Olympus endoscopes (GIF-H260, GIF-H260Z, GIF-HQ290, GIF-H290Z, Olympus Medical Systems, Co., Ltd., Tokyo, Japan) with video processors (EVIS LUCERA CV260/CLV260SL, EVIS LUCERA ELITE CV290/CLV290SL, Olympus Medical Systems, Co., Ltd., Tokyo, Japan).

Two board-certificated pathologists determined the invasion depth of GC according to WHO Classification of Tumors 5th edition in cooperation. We defined D0 as a tumor invasion depth restricted to the mucosa and defined D1 as a tumor invasion depth deeper than mucosa. All the selected images were categorized into D0 (1924 images from 458 patients) and D1 (1711 images from 270 patients) based on the pathologic diagnosis of the resected tissues. These images were then labeled with D0 or D1 and marked with rectangular frames on the lesions by five experienced endoscopists from NJDTH (each of whom had more than 5 years of experience and had performed at least 5000 endoscopic examinations). For the D0 lesions, the whole area of the lesion was marked. But for the D1 lesions, only the region, based on pathological results that potentially invaded deeper than mucosa, was marked. The image marks were finalized only when more than four endoscopists reached a consensus to avoid individual bias. A total of 54 videos that lasted for 10s each were classified into the intramucosal category (M) and the submucosal category (SM) based on the final pathological results.

The whole dataset (3635 images from 728 patients) was divided into training and testing datasets, using random sampling based on patients. The training and testing datasets were as follows: 1) Training dataset: D0: 1798 images from 421 patients, D1: 1609 images from 245 patients, between Jan 2017 and June 2019; 2) Image testing dataset: D0: 126 images from 37 patients, D1: 102 images from 25 patients, between Jan 2017 and June 2019; and 3) Video testing dataset: M: 44 videos of intramucosal lesions from 44 patients, SM: 10 videos of submucosal lesions from 10 patients (**Figure S1**).

### Development and Validation of DCNN Model

In this study, an architecture called Resnet-50 was employed to learn the features of the endoscopy images (22). For most DCNN frameworks, the network layers and the learning ability of the whole network are limited. This limitation is called the Vanishing Gradient problem of DCNN. The Shotcut connection structure enables the DCNN framework to contain more layers, thus effectively alleviating the Vanishing Gradient problem of DCNN. Resnet-50 is a classical framework and most widely employed in the Resnet family to solve complex image classification tasks (**Figure S2**). During the DCNN training process, the parameters of the neurons in the network were initially set to random values. For each input annotated image, the output was computed by the DCNN and compared with the annotation. The parameters of this mathematical function were then modified slightly to decrease the error of the output. The same process was then repeated multiple times for every image in the training set.

### Evaluation of DCNN Model and Comparing With the Endoscopists

Firstly, we evaluated our DCNN model’s performance to diagnose intramucosal GC in the testing datasets described above. Then, 20 endoscopists participated in the following assessment in two groups: (1) novices: 14 novice endoscopists with less than 2 years of endoscopic experience and no more than 3,000 endoscopic examinations; (2) experts: 6 experienced endoscopists with more than 10 years of endoscopic expertise and at least 8,000 endoscopic examinations (acknowledgments: YW, HMG, TY, 7NNZ; co-authors: MC, GFX). None of the endoscopists participated in the selection and labeling of the image datasets. Two-stage tests were conducted to further evaluate the DCNN model’s assistance with the image and video testing datasets in our testing platform (**Figure S3**). The testing images and videos were all anonymized and randomly mixed before the assessments of endoscopists. For testing 1, each endoscopist was asked to diagnose the testing images and videos independently. A week later, these endoscopists conducted testing independently with the presentation of the DCNN-processed diagnosis. After testing 2, a Grit scale was used to assess the individual personality characteristics with 12 items. These items can be divided into two parts: consistency of interest and perseverance of effort. Each item was scored on a 5-point scale (from 1 to 5). The final score was the summed score divided by 12. Grit scale tests were conducted with a free platform (*Document Star*, https://www.wjx.cn).

### Statistical Analyses

The primary outcome of this study was to evaluate the assistance of AI in improving the diagnostic performance of endoscopists. The area under the ROC curve (AUC) was calculated to assess the diagnostic ability of the DCNN model and endoscopists. The diagnostic performance of endoscopists with or without the DCNN model’s assistance was evaluated and compared with the McNemar test. The diagnostic time was analyzed with Wilcoxon rank tests between groups with or without the DCNN model’s assistance. The Grit scale scores were analyzed using correlations and linear regression analyses. For all the tests mentioned, a *p-value* of 0.05 was regarded as statistically significant. All statistical analysis and plotting were conducted with R software (version 4.0.2, R Foundation for Statistical Computing, Vienna, Austria) in R studio (version 1.3.959, R Studio Co., Boston, MA, USA).

## Results

### Performance of DCNN Model in Image Testing Dataset

**Figure 1:**
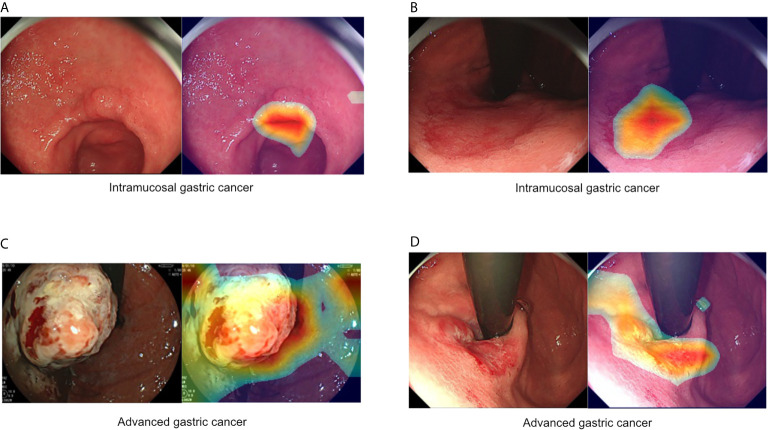
Representative images of intramucosal and advanced gastric cancer. **(A, B)** Intramucosal gastric cancer, original c-WLI (left), and visual representation of the heatmap (right). **(C, D)** Advanced gastric cancer, original c-WLI (left), and visual representation of heatmap (right).

In the testing dataset, the DCNN model could make a diagnosis of D0 from D1 with an AUC of 0.942 [95% confidence interval (CI), 0.915–0.970], a sensitivity of 90.5% (95% CI, 84.1%–95.4%), and a specificity of 85.3% (95% CI, 77.1%–90.9%) (**Table 1** and **Figure 1**). The overall accuracy of our DCNN model was 88.2% (95% CI, 83.3%–91.7%), with a positive predictive value of 88.37% (95% CI, 81.7%–92.8%) and a negative predictive value of 87.88% (95% CI, 81.7%–92.8%) (**Table 1**).

**Table 1:** Performance of the DCNN Model for Diagnosis of Gastric Mucosal Cancer.

	Accuracy, n (%)	Sensitivity, n (%)	Specificity, n (%)	Positive predictive value, n (%)	Negative predictive value, n (%)	Diagnostic time (s)
DCNN-model	88.16 (201/228)	90.48 (114/126)	85.29 (87/102)	88.37 (114/129)	87.88 (87/99)	0.15

### Performance of Endoscopists Without AI Assistance (Test1) and With AI Assistance (Test2) in Image Testing Dataset

**Figure 2:**
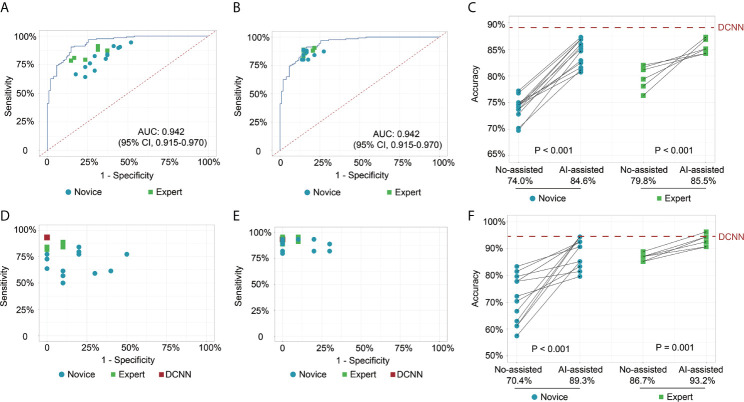
Receiver operating characteristic curves and scatter plots illustrate the DCNN model’s ability and endoscopists in discriminating intramucosal GC. **(A)** Diagnostic performance of DCNN model and endoscopists without the assistance of DCNN model in the image testing datasets; **(B)** Diagnostic performance of DCNN model and endoscopists with the assistance of DCNN model in the image testing datasets; **(C)** Diagnostic accuracy of endoscopists in the subgroup with or without the assistance of DCNN model in the image testing datasets; **(D)** Diagnostic performance of DCNN model and endoscopists without the assistance of DCNN model in the video testing datasets; **(E)** Diagnostic performance of DCNN model and endoscopists with the assistance of DCNN model in the video testing datasets; **(F)** Diagnostic accuracy of endoscopists in the subgroup with or without the assistance of DCNN model in the video testing datasets.

In test 1, the diagnostic performance of the DCNN model was better compared with those of endoscopists in both novice and expert groups (**Figure 2A**). All the endoscopists involved in this study exhibited a lower diagnostic accuracy than the DCNN model (69.7%–82.1% *vs.* 88.2%, P < 0.05) (**Table 2**). For diagnostic concordance, the mean pairwise kappa of the DCNN model was 0.527 (**Figure S4C**). The mean pairwise kappa value of endoscopists varied from 0.430 to 0.629 (**Table S2**).

**Table 2:** Diagnostic Accuracy of Endoscopists with or without the Assistance of DCNN Model.

Endoscopists	No-assistance (Test1)			AI-assistance (Test2)			Test1 *vs.* Test2
	Accuracy			Accuracy			
	n	percent	95% CI	n	percent	95% CI	
Novice (N=14)							
1	166/228	72.8	(66.9–78.7)	195/228	85.5	(81.6–89.4)	< 0.001
2	169/228	74.1	(68.2–80.0)	198/228	86.8	(82.9–90.7)	< 0.001
3	170/228	74.6	(68.7–80.5)	194/228	85.1	(81.2–89.0)	0.001
4	176/228	77.2	(71.3–83.1)	199/228	87.3	(83.4–91.2)	0.001
5	168/228	73.7	(67.8–79.6)	189/228	82.9	(79.0–86.8)	0.007
6	159/228	69.7	(63.8–75.6)	193/228	84.6	(80.8–88.6)	< 0.001
7	170/228	74.6	(68.7–80.5)	198/228	86.8	(82.9–90.7)	< 0.001
8	175/228	76.8	(70.9–82.7)	198/228	86.8	(82.9–90.7)	0.004
9	171/228	75.0	(69.1–80.9)	186/228	81.6	(75.7–87.5)	0.015
10	170/228	74.6	(68.7–80.5)	198/228	86.8	(82.9–90.7)	< 0.001
11	170/228	74.6	(68.7–80.5)	188/228	82.5	(76.6–88.4)	0.021
12	168/228	73.7	(67.8–79.6)	196/228	86.0	(82.1–89.9)	< 0.001
13	170/228	74.6	(68.7–80.5)	184/228	80.7	(74.8–86.6)	0.022
14	160/228	70.2	(64.3–76.1)	185/228	81.1	(75.2–87.0)	< 0.001
Expert endoscopists (N=6)							
1	186/228	81.6	(75.7–87.5)	194/228	85.1	(81.2–89.0)	0.153
2	181/228	79.4	(73.5–85.3)	194/228	85.1	(73.9–85.7)	0.061
3	187/228	82.0	(76.2–88.0)	192/228	84.2	(80.3–88.1)	0.473
4	174/228	76.3	(70.4–82.2)	198/228	86.8	(82.9–90.7)	< 0.001
5	185/228	81.1	(75.2–87.0)	192/228	84.2	(80.3–88.1)	0.248
6	178/228	78.1	(72.2–84.0)	199/228	87.3	(83.4–91.2)	0.005

95% CI, 95% confidence interval.

In test 2, the performance of endoscopists was improved significantly with the DCNN model’s assistance (**Figure 2B**). However, the increase of diagnostic accuracy varied between groups (**Table 2**). All the fourteen novice endoscopists showed significantly increased diagnostic accuracy with the use of the DCNN model (69.7%–77.2% *vs.* 80.7%–87.3%, P < 0.05), while two of six expert endoscopists yielded statistically improved accuracy with the assistance (P < 0.05). Notably, none of the enrolled endoscopists achieved higher accuracy than the DCNN model (**Figure 2C**). The diagnostic accuracy of novice endoscopists was significantly lower than that of expert endoscopists without the DCNN model’s assistance (P < 0.01) (**Figure 2C**). Using the DCNN model, the accuracy of the novice group was comparable to that of the expert group (**Figure 2C**, P = 0.95). For sensitivity, three novices and two experts achieved significantly higher sensitivity with the DCNN model’s assistance (**Table S3** and **Figure S4A**). For specificity, 10 novices and two experts showed significantly increased specificity using the DCNN model (**Table S3** and **Figure S4B**). For expert group, the diagnostic accuracy, sensitivity and specificity were increased significantly with the DCNN model’s assistance (accuracy, 79.8% vs 85.5%, P < 0.001, **Figure 2C**; sensitivity, 84.3% vs 87.4%, P = 0.018; specificity, 74.2% vs 83.0%, P < 0.001; **Table S4**). For the novice group, the diagnostic accuracy, sensitivity and specificity also were elevated remarkably with the DCNN model’s assistance (accuracy, 74.0% vs 84.6%, P < 0.001, **Figure 2C**; sensitivity, 81.1% vs 85.7%, P = 0.018; specificity, 65.2% vs 83.3%, P < 0.001; **Table S4**). As to concordance, the mean pairwise kappa of the DCNN model was 0.861 (**Figure S4D**). The mean pairwise kappa value of endoscopists increased significantly using the DCNN model and varied from 0.660 to 0.861 (**Table S5**).

The diagnostic time of the DCNN model was 0.15 seconds per image, which was much shorter than those of endoscopists (**Table 3**). With the DCNN model’s assistance, the overall diagnostic time of endoscopists shortened significantly (4.35 *vs.* 3.01, P = 0.03). Notably, the diagnostic time of endoscopists was reduced statistically in the novice group (5.09 *vs.* 3.12, P = 0.02) with the DCNN model’s assistance. However, the diagnostic time of experts was marginally increased with the DCNN model (2.62 *vs.* 2.76, P =0.64).

**Table 3:** Diagnostic time of Endoscopists with or without the Assistance of AI.

Diagnostic time (s)	No-assistance (Test1)	AI-assistance (Test2)	P-value
DCNN model	0.15	0.15	–
Overall	4.35 ± 3.02	3.01 ± 1.66	0.03
Novice	5.09 ± 3.33	3.12 ± 1.90	0.02
Expert	2.62 ± 0.77	2.76 ± 0.99	0.64

### Performance of Endoscopists Without AI Assistance (Test1) and With AI Assistance (Test2) in Video Testing Dataset

To further explore the assistance of the DCNN model in a real-time clinical setting, we evaluated the performance of endoscopists with or without the DCNN model’s assistance with 54 endoscopic videos (**Figures 2D, E**). The DCNN model showed a better performance in the video datasets with a sensitivity of 93.2%, a specificity of 100.0%, and an accuracy of 94.4% (**Table S6**). For expert endoscopists, the diagnostic accuracy and sensitivity increased significantly with the assistance of the DCNN model (accuracy, 86.7% vs 93.2%, P = 0.001, **Figure 2F**; sensitivity, 85.2% vs 92.4%, P = 0.002, **Table S6**). But the specificity showed marginal improvement (93.3% *vs.* 96.7%, P = 0.617, **Table S6**). For novice endoscopists, the diagnostic accuracy, sensitivity and specificity increased remarkably with the assistance of the DCNN model (accuracy, 70.4% vs 89.3%, P < 0.001, **Figure 2F**; sensitivity, 67.7% vs 88.6%, P < 0.001; specificity, 82.1% vs 92.1%, P = 0.008; **Table S6**).

### Personality Traits and Performance of Endoscopists

**Figure 3:**
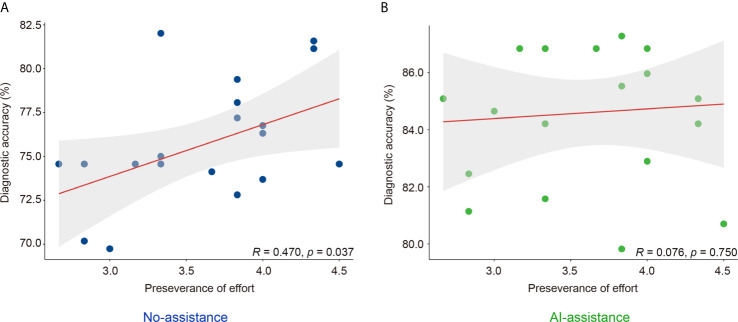
Correlation of perseverance of effort and diagnostic accuracy of endoscopists with **(B)** or without **(A)** the DCNN model’s assistance.

Grit scale reflects the ability of individuals to maintain focus (consistency of interest) and persevering for long-term goals (perseverance of effort) (23). The correlation between the personality traits and the diagnostic accuracy was analyzed with or without the DCNN model’s assistance. As is shown here, the correlation between grit score and diagnostic accuracy was marginal with or without the assistance of DCNN (r = 0.178, P = 0.452 *vs.* r = 0.145, P = 0.541, **Table 4** and **Figure S5**). The correlation between the scores for the consistency of interest and the diagnostic accuracy was also not significant with or without the assistance of DCNN (r = -0.122, P = 0.609 *vs.* r = 0.145, P = 0.541, **Table 4** and **Figure S5**). Intestinally, the results showed that a moderate correlation between the scores for perseverance of effort and the diagnostic accuracy existed when endoscopists made the diagnosis without the DCNN’s assistance (r = 0.470, P = 0.037, **Table 4** and **Figure 3**). Notably, there was no significant correlation between the scores for the perseverance of effort and diagnostic accuracy when the endoscopists were assisted with the DCNN (r = 0.076, P = 0.750, **Table 4** and **Figure 3**).

**Table 4:** Correlation between Grit Score and Diagnostic Accuracy.

	Score		Diagnostic accuracy			
			No-assistance (Test1)		AI-assistance (Test2)	
	Mean ± sd	IQR	Correlation, r	P-value	Correlation, r	P-value
Grit score	3.546 ± 0.479	3.083–3.917	0.178	0.452	0.145	0.541
Consistency of interest	3.458 ± 0.677	3.167–3.833	-0.122	0.609	0.145	0.541
Perseverance of effort	3.633 ± 0.540	3.292–4.000	0.470	0.037	0.076	0.750

Sd, standard deviation; IQR, interquartile range.

## Discussion

In this study, we developed a DCNN model to assist endoscopists in making accurate intramucosal GC diagnoses. The DCNN model showed satisfactory diagnostic performance in discriminating intramucosal GC from advanced lesions. We investigated the assistance of the DCNN model on the improvement of diagnostic performance of endoscopists. With the DCNN model’s assistance, the diagnostic accuracy of endoscopists increased statistically in both novice and expert groups. The diagnostic agreement among endoscopists also increased from a moderate level to a substantial level with the DCNN model. The diagnostic time was significantly shortened with the DCNN model’s assistance, especially in the novice group.

Operational resection is the only curative therapy for GC, but this therapy can only be adopted in GC patients at an early stage. However, most patients are diagnosed at an advanced stage and are ineligible for curative resection. Previous studies reported that while the 5-year survival rate of advanced GC remained less than 25%, the 5-year survival rate of intramucosal GC exceeded 95% (4, 5). Therefore, it is exceptionally crucial to accurately differentiate intramucosal GC from advanced lesions for preoperative evaluation and determining the optimal treatment (8).

C-WLI was the main-used modality to predict intramucosal GC, with its accuracy ranging from 70% to 85%. Although the diagnostic performance was comparable to other modalities like EUS or ME-NBI, 15% of cases would be underestimated or overestimated (10, 12, 14, 15). Moreover, since endoscopic examinations were relatively subjective, the interobserver agreement varied significantly amongst endoscopists with different expertise (24). AI-assisted diagnostic devices may help improve the relatively low accuracy and interobserver agreement and reduce the time and effort required to master these methodologies. Three preliminary studies have reported that AI showed a better performance in the diagnosis of intramucosal GC than C-WLI, with the accuracy ranging from 73.0% to 94.5% (18, 20, 21). Here, we developed a DCNN model with a robust performance in discriminating intramucosal GC from advanced lesions with an AUC value of 0.942. The accuracy, sensitivity, and specificity of the DCNN model were 88.2%, 90.5%, and 85.3%, respectively. Consistent with the previously reported DCNN systems, our model showed satisfactory diagnostic performance. However, these preliminary studies mainly focused on developing and validating AI models to diagnose intramucosal GC. Rare studies were conducted to evaluate the assistance of AI models in improving the diagnostic performance of endoscopists. This is extremely important since AI models cannot make the final diagnosis considering safety, accountability, and ethics despite having higher diagnostic accuracy, sensitivity, and specificity than expert endoscopists. Therefore, we further evaluated the assistant role of the DCNN model on the diagnostic performance of endoscopists.

This study showed that endoscopists could benefit significantly from AI assistance in three areas. Firstly, novices achieved considerable improvement in diagnostic performance, which was approximately the same as experts with AI assistance. This improvement significantly reduced the threshold for novices predicting intramucosal GC, which may help these novice endoscopists predict more intramucosal GC during endoscopic examinations. Technologies of visualization were used to locate the intramucosal GC lesions in the images, enabling endoscopists to understand these lesions (**Video 1**) intuitively. Moreover, consistent with previous studies, the diagnostic specificity of intramucosal GC in inexperienced endoscopists was relatively low without effective training (10, 14). Notably, low specificity can be catastrophic since it indicates too many advanced GC lesions being underestimated as intramucosal GC. Therefore, improvement in specificity is essential for optimizing the benefit for patients. Herein, we noticed a significant increase of specificity in novice endoscopists with AI assistance, which may reduce the under-diagnosis rate in clinical practice. Secondly, the interobserver agreement among endoscopists was elevated significantly with the DCNN model’s assistance. Several studies have reported relatively low interobserver agreement of novice endoscopists in diagnosing gastric lesions during endoscopic examinations (25). In this study, we noticed that the interobserver agreement of novice endoscopists was comparable with that of experienced endoscopists with AI assistance. The high agreement reduced the discrepancy in diagnosis and promoted homogenization of diagnostic performance, thus alleviating the diagnostic disputes observed in China. Thirdly, diagnostic duration was statistically reduced in the novice group. This indicates that the DCNN model may help endoscopists with limited training increase their diagnostic efficiency. However, we noticed a slightly longer diagnostic time in expert endoscopists. This may be induced by time lags arising from the inconsistencies between the diagnoses made by the DCNN model and the experts. While novice endoscopists tend to accept the diagnosis of the DCNN model, the experts tend to think it over when they encounter inconsistent diagnoses made by the DCNN.

To gain competence in endoscopic procedures, endoscopists need to practice a substantial amount to reach the threshold number (26). With AI assistance, novice endoscopists achieved comparable diagnostic performance with experts without much additional effort. Additionally, several studies have used Grit Scale tests to evaluate the perseverance and interest for long-time goals (23, 27, 28). Higher grit scores were associated with better performance in multiple settings, including medical school and residency training (27, 28). A previous study indicated that higher grit, significantly higher consistency of interest, was associated with the flexible acceptance of AI assistance (29). However, we noticed that a higher score of effort was correlated with diagnostic accuracy without AI assistance. With AI assistance, the correlation between the perseverance of effort and diagnostic accuracy was diminished. This indicates that AI assistance may reduce the threshold number of procedures required by endoscopists to gain competence. However, this also brings up the point that the novices may begin to rely too much on AI assistance, reducing their ability to make independent diagnoses. Therefore, further investigations are required to evaluate the effect of AI assistance on independent diagnosis ability in endoscopists.

This study has several limitations. Firstly, the DCNN model cannot be applied to poor-quality images, and we excluded these poor-quality images, including images with less insufflation of air, halation, defocus, blurs. We are collecting these poor-quality images and developing an AI classification model to discriminate between poor-quality and high-quality images to solve this issue. Secondly, the training and testing datasets are from one retrospective dataset, which cannot rule out selection bias. As the testing dataset was randomly selected from the retrospective dataset, the excellent performance of the DCNN model in this independent dataset partly demonstrated the potential of this DCNN model. However, the performance and generalizability remained to be evaluated in other prospective datasets. Thirdly, this is a retrospective study, and the excellent performance of the DCNN system may not reflect the clinical application in the real world. Here, we used 54 videos to assess the real-time performance of AI and evaluate the AI assistance on endoscopists to imitate the actual clinical settings. This may partly demonstrate a good result of AI assistance on the performance of endoscopists. But prospective randomized controlled trials are needed to validate the results in actual clinical settings. Fourth, we only included images with histologically proven malignancy, indicating the system could not be used to differentiate malignant lesions from non-cancer mucosa. We have established an AI system in detecting early gastric cancer from non-cancer mucosa in our previous report (30). The two systems can be used together to detect early gastric cancer lesions from non-cancer mucosa first and then differentiate intramucosal GC from advanced lesions, thus may facilitate the endoscopic treatment of GC.

## Conclusion

In conclusion, we developed and validated an AI-assisted system that could predict intramucosal GC with high accuracy and short duration. We found that AI assistance helped novice endoscopists achieve comparable diagnostic accuracy and duration with expert endoscopists with minimal training or effort. In the future, more studies are needed to examine the effect of AI-assisted systems on the ability of novice endoscopists to establish independent diagnoses.

## Data Availability Statement

The datasets used and analyzed during the study are available from the corresponding author on reasonable request approved by the IRB of Nanjing University Medical School Affiliated Drum Tower Hospital (XP.Z. zouxp@nju.edu.cn).

## Ethics Statement

The study design was reviewed and approved by the Medical Ethics Committee at each institution (NJDTH, IRB no. 2020-026-01; RHWU, WDRY2019-K091). Informed consent was waived given the use of only retrospectively deidentified endoscopic images.

## Author Contributions

XZ, GX, and HY conceived and designed the study. DT, JZ, LW, MN, RL, XC, XH, and LZ contributed to the acquisition of data. DT, MC, and XD contributed to analysis and interpretation of data. HY supervised the construction of deep learning algorithms. DT, JZ, GX, and XZ drafted and reviewed the manuscript. SH reviewed the manuscript and conducted language editing. XZ supported the project. All authors contributed to the article and approved the submitted version.

## Funding

This project was supported by the National Natural Science Foundation of China (Grant Nos. 81672935, 81871947), Jiangsu Clinical Medical Center of Digestive System Diseases and Gastrointestinal Cancer (Grant No. YXZXB2016002), and the Nanjing Science and technology development Foundation (Grant No. 2017sb332019). The funders were not involved in the study design, data collection, analysis, or manuscript preparation.

## Conflict of Interest

The authors declare that the research was conducted in the absence of any commercial or financial relationships that could be construed as a potential conflict of interest.

## Abbreviations

AI, Artificial intelligence; WLI, White light imaging; NBI, Narrow band imaging; PPV, Positive predictive value; NPV, Negative predictive value; ESD, Endoscopic submucosal dissection; DCNN, Deep convolutional neural network; GC, Gastric cancer; LNM, Lymph node metastasis; EUS, Endoscopic ultrasound; ME, Magnifying endoscopy; NBI, Narrow-band imaging; BLI, Blue laser imaging; C-WLI, Conventional white-light imaging.
