# Supplementary material for: A Novel Model Based on Deep Convolutional Neural Network Improves Diagnostic Accuracy of Intramucosal Gastric Cancer (With Video)
Source: Front Oncol. 2021 Apr 20;11:622827. doi: 10.3389/fonc.2021.622827 (PMC8095170; doi:10.3389/fonc.2021.622827)
Supplement: Supplementary file 1 [file DataSheet_1.docx]

**Supplementary materials**

**Figure Legends and Tables**

Figure S1 Workflow for this study

Figure S2 The architecture and workflow of the DCNN model.

Figure S3 Representative images of our testing platform.

Figure S4 Performance of endoscopists with or without using the DCNN model. Sensitivity (A) and specificity (B) of endoscopists with or without the assistance of the DCNN model. (C) The mean pairwise kappa value and ranking of endoscopists without using the DCNN model. (D) The mean pairwise kappa value and ranking of endoscopists with the assistance of the DCNN model.

Figure S5 Correlation of personal traits and diagnostic accuracy. (A) Correlation of Grit score and diagnostic accuracy of endoscopists with (right) or without (left) the assistance of DCNN model. (B) Consistency of interest and diagnostic accuracy of endoscopists with (right) or without (left) the assistance of DCNN model.

Table S1 Baseline characteristics of datasets

| Characteristics | Development (n = 666) | Test (n = 62) | Video (n = 54) |
| --- | --- | --- | --- |
| Age (yr), mean (range) | 63.6 (31–84) | 62.8 (37–80) | 63.5 (53–74) |
| Sex, n (%) |  |  |  |
| Female | 475 (71.3) | 44 (71.0) | 38 (70.4%) |
| Male | 191 (28.7) | 18 (29.0) | 16 (29.6%) |
| Size (cm), mean (range) | 1.9 (0.2–8.0) | 2.1 (0.2–7.1) | 1.9 (0.4–4.5) |
| Location, n (%) |  |  |  |
| Cardiac | 243 (36.5) | 23 (37.1) | 11 (20.4%) |
| Gastric fundus | 7 (1.1) | 6 (9.6) | 0 |
| Gastric body | 95 (14.3) | 9 (14.5) | 0 |
| Angulus | 126 (18.9) | 12 (19.4) | 14 (25.9%) |
| Antrum | 195 (29.3) | 12 (19.4) | 29 (53.7%) |
| Macroscopic type, n (%) |  |  |  |
| I | 6 (0.9) | 5 (8.1) | 2 (3.7%) |
| IIa | 146 (21.9) | 13 (21.0) | 4 (7.4%) |
| IIb | 111 (16.7) | 10 (16.1) | 1 (1.9%) |
| IIc | 270 (40.5) | 22 (35.5) | 31 (57.4%) |
| IIa+IIc | 95 (14.3) | 9 (14.5) | 11 (20.4%) |
| IIc+IIa | 12 (1.8) | 2 (3.2) | 2 (3.7%) |
| IIb+IIc | 25 (3.8) | 1 (1.6) | 2 (3.7%) |
| III | 1 (0.1) | 0 | 1 (1.9%) |
| Degree of differentiation, n (%) |  |  |  |
| Differentiated | 483(72.5) | 47 (75.8) | 36 (66.7%) |
| Undifferentiated | 20 (3.0) | 1 (1.6) | 13 (24.1%) |
| Mixed | 163 (24.5) | 14 (22.6) | 5 (9.3%) |
| Invasion depth, n (%) |  |  |  |
| M | 421 (63.2) | 37 (59.7) | 44 (81.5%) |
| SM | 245 (36.8) | 25 (40.3) | 10 (18.5%) |

Table S2 Interobserver Agreement of Endoscopists without the assistance of AI

| Kappa value | | | | | | | | | | | | | | | | | | | | | |  |  |
| --- | --- | --- | --- | --- | --- | --- | --- | --- | --- | --- | --- | --- | --- | --- | --- | --- | --- | --- | --- | --- | --- | --- | --- |
| Novice | | | | | | | | | | | | | | | | Expert | | | | | |  | AI |
|  | 1 | 2 | 3 | 4 | 5 | 6 | 7 | 8 | 9 | 10 | 11 | 12 | 13 | 14 |  | 1 | 2 | 3 | 4 | 5 | 6 |  | AI |
| AI | 0.498 | 0.468 | 0.488 | 0.517 | 0.427 | 0.365 | 0.513 | 0.479 | 0.514 | 0.525 | 0.455 | 0.500 | 0.487 | 0.437 |  | 0.579 | 0.558 | 0.568 | 0.547 | 0.575 | 0.557 |  | 1.000 |
| Novice | | | | | | | | | | | | | | | | | | | | | |  |  |
| 1 | 1.000 | 0.676 | 0.523 | 0.672 | 0.434 | 0.525 | 0.666 | 0.705 | 0.670 | 0.637 | 0.507 | 0.568 | 0.655 | 0.540 |  | 0.649 | 0.713 | 0.584 | 0.663 | 0.636 | 0.696 |  | 0.498 |
| 2 | 0.676 | 1.000 | 0.446 | 0.550 | 0.376 | 0.398 | 0.558 | 0.612 | 0.552 | 0.582 | 0.515 | 0.466 | 0.599 | 0.478 |  | 0.570 | 0.644 | 0.539 | 0.611 | 0.640 | 0.614 |  | 0.468 |
| 3 | 0.523 | 0.446 | 1.000 | 0.548 | 0.393 | 0.293 | 0.570 | 0.538 | 0.430 | 0.608 | 0.435 | 0.651 | 0.647 | 0.395 |  | 0.386 | 0.523 | 0.384 | 0.504 | 0.477 | 0.455 |  | 0.488 |
| 4 | 0.672 | 0.550 | 0.548 | 1.000 | 0.414 | 0.472 | 0.577 | 0.559 | 0.653 | 0.567 | 0.490 | 0.538 | 0.641 | 0.559 |  | 0.544 | 0.605 | 0.602 | 0.630 | 0.603 | 0.608 |  | 0.517 |
| 5 | 0.434 | 0.376 | 0.393 | 0.414 | 1.000 | 0.266 | 0.437 | 0.401 | 0.400 | 0.376 | 0.457 | 0.334 | 0.360 | 0.458 |  | 0.386 | 0.428 | 0.432 | 0.405 | 0.430 | 0.409 |  | 0.427 |
| 6 | 0.525 | 0.398 | 0.293 | 0.472 | 0.266 | 1.000 | 0.459 | 0.489 | 0.529 | 0.413 | 0.290 | 0.368 | 0.481 | 0.449 |  | 0.465 | 0.467 | 0.388 | 0.443 | 0.468 | 0.521 |  | 0.365 |
| 7 | 0.666 | 0.558 | 0.570 | 0.577 | 0.437 | 0.459 | 1.000 | 0.757 | 0.507 | 0.746 | 0.454 | 0.656 | 0.666 | 0.433 |  | 0.579 | 0.671 | 0.476 | 0.581 | 0.554 | 0.605 |  | 0.513 |
| 8 | 0.705 | 0.612 | 0.538 | 0.559 | 0.401 | 0.489 | 0.757 | 1.000 | 0.566 | 0.784 | 0.479 | 0.750 | 0.721 | 0.493 |  | 0.588 | 0.690 | 0.483 | 0.674 | 0.685 | 0.628 |  | 0.479 |
| 9 | 0.670 | 0.552 | 0.430 | 0.653 | 0.400 | 0.529 | 0.507 | 0.566 | 1.000 | 0.575 | 0.499 | 0.497 | 0.610 | 0.499 |  | 0.640 | 0.588 | 0.577 | 0.650 | 0.641 | 0.585 |  | 0.514 |
| 10 | 0.637 | 0.582 | 0.608 | 0.567 | 0.376 | 0.413 | 0.746 | 0.784 | 0.575 | 1.000 | 0.417 | 0.760 | 0.709 | 0.449 |  | 0.579 | 0.621 | 0.456 | 0.683 | 0.694 | 0.601 |  | 0.525 |
| 11 | 0.507 | 0.515 | 0.435 | 0.490 | 0.457 | 0.290 | 0.454 | 0.479 | 0.499 | 0.417 | 1.000 | 0.380 | 0.382 | 0.438 |  | 0.509 | 0.568 | 0.552 | 0.524 | 0.497 | 0.543 |  | 0.455 |
| 12 | 0.568 | 0.466 | 0.651 | 0.538 | 0.334 | 0.368 | 0.656 | 0.750 | 0.497 | 0.760 | 0.380 | 1.000 | 0.691 | 0.339 |  | 0.439 | 0.587 | 0.399 | 0.607 | 0.578 | 0.467 |  | 0.500 |
| 13 | 0.655 | 0.599 | 0.647 | 0.641 | 0.360 | 0.481 | 0.666 | 0.721 | 0.610 | 0.709 | 0.382 | 0.691 | 1.000 | 0.502 |  | 0.509 | 0.658 | 0.491 | 0.681 | 0.633 | 0.545 |  | 0.487 |
| 14 | 0.540 | 0.478 | 0.395 | 0.559 | 0.458 | 0.449 | 0.433 | 0.493 | 0.499 | 0.449 | 0.438 | 0.339 | 0.502 | 1.000 |  | 0.456 | 0.478 | 0.499 | 0.486 | 0.495 | 0.542 |  | 0.437 |
| Expert | | | | | | | | | | | | | | | | | | | | | |  |  |
| 1 | 0.649 | 0.570 | 0.386 | 0.544 | 0.386 | 0.465 | 0.579 | 0.588 | 0.640 | 0.579 | 0.509 | 0.439 | 0.509 | 0.456 |  | 1.000 | 0.658 | 0.658 | 0.579 | 0.640 | 0.667 |  | 0.579 |
| 2 | 0.713 | 0.644 | 0.523 | 0.605 | 0.428 | 0.467 | 0.671 | 0.690 | 0.588 | 0.621 | 0.568 | 0.587 | 0.658 | 0.478 |  | 0.658 | 1.000 | 0.574 | 0.668 | 0.678 | 0.632 |  | 0.558 |
| 3 | 0.584 | 0.539 | 0.384 | 0.602 | 0.432 | 0.388 | 0.476 | 0.483 | 0.577 | 0.456 | 0.552 | 0.399 | 0.491 | 0.499 |  | 0.658 | 0.574 | 1.000 | 0.600 | 0.645 | 0.586 |  | 0.568 |
| 4 | 0.663 | 0.611 | 0.504 | 0.630 | 0.405 | 0.443 | 0.581 | 0.674 | 0.650 | 0.683 | 0.524 | 0.607 | 0.681 | 0.486 |  | 0.579 | 0.668 | 0.600 | 1.000 | 0.702 | 0.604 |  | 0.547 |
| 5 | 0.636 | 0.640 | 0.477 | 0.603 | 0.430 | 0.468 | 0.554 | 0.685 | 0.641 | 0.694 | 0.497 | 0.578 | 0.633 | 0.495 |  | 0.640 | 0.678 | 0.645 | 0.702 | 1.000 | 0.559 |  | 0.575 |
| 6 | 0.696 | 0.614 | 0.455 | 0.608 | 0.409 | 0.521 | 0.605 | 0.628 | 0.585 | 0.601 | 0.543 | 0.467 | 0.545 | 0.542 |  | 0.667 | 0.632 | 0.586 | 0.604 | 0.559 | 1.000 |  | 0.557 |

Table S3 Sensitivity and Specificity Based on Endoscopic Experience with or without the Assistance of AI

| Endoscopists | Sensitivity | | |  | Specificity | | |
| --- | --- | --- | --- | --- | --- | --- | --- |
|  | No-assistance (Test1), % | AI-assistance (Test2), % | Test1 vs. Test2 |  | No-assistance (Test1), % | AI-assistance (Test2), % | Test1 vs. Test2 |
| Novice (N=14) | | | | | | | |
| 1 | 80.2 | 87.3 | 0.110 |  | 63.7 | 83.3 | < 0.001 |
| 2 | 83.3 | 88.1 | 0.522 |  | 62.7 | 85.3 | < 0.001 |
| 3 | 89.7 | 86.5 | 0.394 |  | 55.9 | 83.3 | < 0.001 |
| 4 | 82.5 | 88.9 | 0.153 |  | 70.6 | 85.3 | 0.002 |
| 5 | 66.7 | 80.2 | 0.005 |  | 82.4 | 86.3 | 0.522 |
| 6 | 64.3 | 84.1 | < 0.001 |  | 76.5 | 85.3 | 0.150 |
| 7 | 84.1 | 88.1 | 0.424 |  | 62.7 | 85.3 | < 0.001 |
| 8 | 91.3 | 88.1 | 0.522 |  | 58.8 | 85.3 | < 0.001 |
| 9 | 76.2 | 84.1 | 0.055 |  | 73.5 | 78.4 | 0.228 |
| 10 | 89.7 | 88.1 | 0.823 |  | 55.9 | 85.3 | < 0.001 |
| 11 | 73.0 | 80.2 | 0.151 |  | 76.5 | 85.3 | 0.095 |
| 12 | 94.4 | 88.1 | 0.080 |  | 48.0 | 83.3 | < 0.001 |
| 13 | 90.5 | 87.3 | 0.343 |  | 54.9 | 72.5 | < 0.001 |
| 14 | 69.8 | 80.2 | 0.037 |  | 70.6 | 82.4 | 0.010 |
| Expert (N=6) | | | | | | | |
| 1 | 78.6 | 85.7 | 0.027 |  | 85.3 | 84.3 | 1.000 |
| 2 | 88.1 | 90.5 | 0.628 |  | 68.6 | 78.4 | 0.066 |
| 3 | 81.0 | 83.3 | 0.689 |  | 83.3 | 85.3 | 0.683 |
| 4 | 87.3 | 88.1 | 1.000 |  | 62.7 | 85.3 | < 0.001 |
| 5 | 91.3 | 88.1 | 0.387 |  | 68.6 | 79.4 | 0.010 |
| 6 | 79.4 | 88.9 | 0.031 |  | 76.5 | 85.3 | 0.110 |

Table S4 Assistance of the DCNN model on the performance of endoscopists with the image testing dataset

|  | DCNN | No-assistance | AI-assistance | P value |
| --- | --- | --- | --- | --- |
| Expert |  |  |  |  |
| Accuracy (%, 95% CI) | 88.2 (83.3–91.7) | 79.8 (77.5–81.8) | 85.5 (83.5–87.2) | < 0.001 |
| Sensitivity (%, 95% CI) | 90.5 (84.1–95.4) | 84.3 (81.7–86.9) | 87.4 (85.1–89.8) | 0.018 |
| Specificity (%, 95% CI) | 85.3 (77.1–90.9) | 74.2 (70.7–77.7) | 83.0 (80.0–86.0) | < 0.001 |
| Novice |  |  |  |  |
| Accuracy (%, 95% CI) | 88.2 (83.3–91.7) | 74.0 (72.4–75.5) | 84.6 (83.3–85.8) | < 0.001 |
| Sensitivity (%, 95% CI) | 90.5 (84.1–95.4) | 81.1 (79.3–82.9) | 85.7 (84.0–87.3) | < 0.001 |
| Specificity (%, 95% CI) | 85.3 (77.1–90.9) | 65.2 (62.7–67.7) | 83.3 (81.4–85.3) | < 0.001 |

Table S5 Interobserver Agreement of Endoscopists with the assistance of AI

| Kappa value | | | | | | | | | | | | | | | | | | | | | |  |  |
| --- | --- | --- | --- | --- | --- | --- | --- | --- | --- | --- | --- | --- | --- | --- | --- | --- | --- | --- | --- | --- | --- | --- | --- |
| Novice | | | | | | | | | | | | | | | | Expert | | | | | |  | AI |
|  | 1 | 2 | 3 | 4 | 5 | 6 | 7 | 8 | 9 | 10 | 11 | 12 | 13 | 14 |  | 1 | 2 | 3 | 4 | 5 | 6 |  | AI |
| AI | 0.938 | 1.000 | 0.947 | 0.920 | 0.833 | 0.956 | 1.000 | 1.000 | 0.680 | 1.000 | 0.807 | 0.982 | 0.623 | 0.744 |  | 0.734 | 0.660 | 0.735 | 0.787 | 0.768 | 0.973 |  | 1.000 |
| Novice | | | | | | | | | | | | | | | | | | | | | |  |  |
| 1 | 1.000 | 0.938 | 0.885 | 0.893 | 0.807 | 0.912 | 0.938 | 0.938 | 0.706 | 0.938 | 0.763 | 0.920 | 0.649 | 0.736 |  | 0.743 | 0.650 | 0.779 | 0.760 | 0.777 | 0.911 |  | 0.938 |
| 2 | 0.938 | 1.000 | 0.947 | 0.920 | 0.833 | 0.956 | 1.000 | 1.000 | 0.680 | 1.000 | 0.807 | 0.982 | 0.623 | 0.744 |  | 0.734 | 0.660 | 0.735 | 0.787 | 0.768 | 0.973 |  | 1.000 |
| 3 | 0.885 | 0.947 | 1.000 | 0.885 | 0.833 | 0.903 | 0.947 | 0.947 | 0.662 | 0.947 | 0.789 | 0.929 | 0.605 | 0.709 |  | 0.717 | 0.660 | 0.700 | 0.752 | 0.732 | 0.920 |  | 0.947 |
| 4 | 0.893 | 0.920 | 0.885 | 1.000 | 0.772 | 0.912 | 0.920 | 0.920 | 0.724 | 0.920 | 0.763 | 0.902 | 0.703 | 0.771 |  | 0.743 | 0.704 | 0.744 | 0.831 | 0.795 | 0.911 |  | 0.920 |
| 5 | 0.807 | 0.833 | 0.833 | 0.772 | 1.000 | 0.807 | 0.833 | 0.833 | 0.605 | 0.833 | 0.728 | 0.816 | 0.552 | 0.684 |  | 0.658 | 0.569 | 0.640 | 0.728 | 0.657 | 0.824 |  | 0.833 |
| 6 | 0.912 | 0.956 | 0.903 | 0.912 | 0.807 | 1.000 | 0.956 | 0.956 | 0.691 | 0.956 | 0.763 | 0.938 | 0.671 | 0.754 |  | 0.762 | 0.654 | 0.745 | 0.815 | 0.779 | 0.929 |  | 0.956 |
| 7 | 0.938 | 1.000 | 0.947 | 0.920 | 0.833 | 0.956 | 1.000 | 1.000 | 0.680 | 1.000 | 0.807 | 0.982 | 0.623 | 0.744 |  | 0.734 | 0.660 | 0.735 | 0.787 | 0.768 | 0.973 |  | 1.000 |
| 8 | 0.938 | 1.000 | 0.947 | 0.920 | 0.833 | 0.956 | 1.000 | 1.000 | 0.680 | 1.000 | 0.807 | 0.982 | 0.623 | 0.744 |  | 0.734 | 0.660 | 0.735 | 0.787 | 0.768 | 0.973 |  | 1.000 |
| 9 | 0.706 | 0.680 | 0.662 | 0.724 | 0.605 | 0.691 | 0.680 | 0.680 | 1.000 | 0.680 | 0.596 | 0.662 | 0.658 | 0.674 |  | 0.769 | 0.677 | 0.770 | 0.716 | 0.785 | 0.689 |  | 0.680 |
| 10 | 0.938 | 1.000 | 0.947 | 0.920 | 0.833 | 0.956 | 1.000 | 1.000 | 0.680 | 1.000 | 0.807 | 0.982 | 0.623 | 0.744 |  | 0.734 | 0.660 | 0.735 | 0.787 | 0.768 | 0.973 |  | 1.000 |
| 11 | 0.763 | 0.807 | 0.789 | 0.763 | 0.728 | 0.763 | 0.807 | 0.807 | 0.596 | 0.807 | 1.000 | 0.789 | 0.560 | 0.658 |  | 0.649 | 0.542 | 0.649 | 0.701 | 0.683 | 0.798 |  | 0.807 |
| 12 | 0.920 | 0.982 | 0.929 | 0.902 | 0.816 | 0.938 | 0.982 | 0.982 | 0.662 | 0.982 | 0.789 | 1.000 | 0.640 | 0.727 |  | 0.716 | 0.677 | 0.717 | 0.769 | 0.750 | 0.956 |  | 0.982 |
| 13 | 0.649 | 0.623 | 0.605 | 0.703 | 0.552 | 0.671 | 0.623 | 0.623 | 0.658 | 0.623 | 0.560 | 0.640 | 1.000 | 0.672 |  | 0.696 | 0.616 | 0.681 | 0.713 | 0.673 | 0.649 |  | 0.623 |
| 14 | 0.736 | 0.744 | 0.709 | 0.771 | 0.684 | 0.754 | 0.744 | 0.744 | 0.674 | 0.744 | 0.658 | 0.727 | 0.672 | 1.000 |  | 0.692 | 0.602 | 0.745 | 0.762 | 0.726 | 0.736 |  | 0.744 |
| Expert | | | | | | | | | | | | | | | | | | | | | |  |  |
| 1 | 0.743 | 0.734 | 0.717 | 0.743 | 0.658 | 0.762 | 0.734 | 0.734 | 0.769 | 0.734 | 0.649 | 0.716 | 0.696 | 0.692 |  | 1.000 | 0.679 | 0.841 | 0.770 | 0.840 | 0.743 |  | 0.734 |
| 2 | 0.650 | 0.660 | 0.660 | 0.704 | 0.569 | 0.654 | 0.660 | 0.660 | 0.677 | 0.660 | 0.542 | 0.677 | 0.616 | 0.602 |  | 0.679 | 1.000 | 0.681 | 0.642 | 0.656 | 0.668 |  | 0.660 |
| 3 | 0.779 | 0.735 | 0.700 | 0.744 | 0.640 | 0.745 | 0.735 | 0.735 | 0.770 | 0.735 | 0.649 | 0.717 | 0.681 | 0.745 |  | 0.841 | 0.681 | 1.000 | 0.753 | 0.841 | 0.709 |  | 0.735 |
| 4 | 0.760 | 0.787 | 0.752 | 0.831 | 0.728 | 0.815 | 0.787 | 0.787 | 0.716 | 0.787 | 0.701 | 0.769 | 0.713 | 0.762 |  | 0.770 | 0.642 | 0.753 | 1.000 | 0.839 | 0.796 |  | 0.787 |
| 5 | 0.777 | 0.768 | 0.732 | 0.795 | 0.657 | 0.779 | 0.768 | 0.768 | 0.785 | 0.768 | 0.683 | 0.750 | 0.673 | 0.726 |  | 0.840 | 0.656 | 0.841 | 0.839 | 1.000 | 0.759 |  | 0.768 |
| 6 | 0.911 | 0.973 | 0.920 | 0.911 | 0.824 | 0.929 | 0.973 | 0.973 | 0.689 | 0.973 | 0.798 | 0.956 | 0.649 | 0.736 |  | 0.743 | 0.668 | 0.709 | 0.796 | 0.759 | 1.000 |  | 0.973 |

Table S6 Assistance of the DCNN model on the performance of endoscopists with the video testing dataset

|  | DCNN | No-assistance | AI-assistance | P value |
| --- | --- | --- | --- | --- |
| Expert |  |  |  |  |
| Accuracy (%, 95% CI) | 94.4 (84.9–98.5) | 86.7 (82.6–90.0) | 93.2 (89.9–95.5) | 0.001 |
| Sensitivity (%, 95% CI) | 93.2 (85.7–100.0) | 85.2 (80.9–89.5) | 92.4 (89.2–95.6) | 0.002 |
| Specificity (%, 95% CI) | 100.0 (100.0–100.0) | 93.3 (87.0–99.6) | 96.7 (92.1–100.0) | 0.617 |
| Novice |  |  |  |  |
| Accuracy (%, 95% CI) | 94.4 (84.9–98.5) | 70.4 (67.0–73.5) | 89.3 (86.9–91.3) | < 0.001 |
| Sensitivity (%, 95% CI) | 93.2 (85.7–100.0) | 67.7 (64.0–71.4) | 88.6 (86.1–91.1) | < 0.001 |
| Specificity (%, 95% CI) | 100.0 (100.0–100.0) | 82.1 (75.8–88.5) | 92.1 (87.7–96.6) | 0.008 |
