# Supplementary figures and images for: A Novel Model Based on Deep Convolutional Neural Network Improves Diagnostic Accuracy of Intramucosal Gastric Cancer (With Video)
Source: Front Oncol. 2021 Apr 20;11:622827. doi: 10.3389/fonc.2021.622827 (PMC8095170; doi:10.3389/fonc.2021.622827)

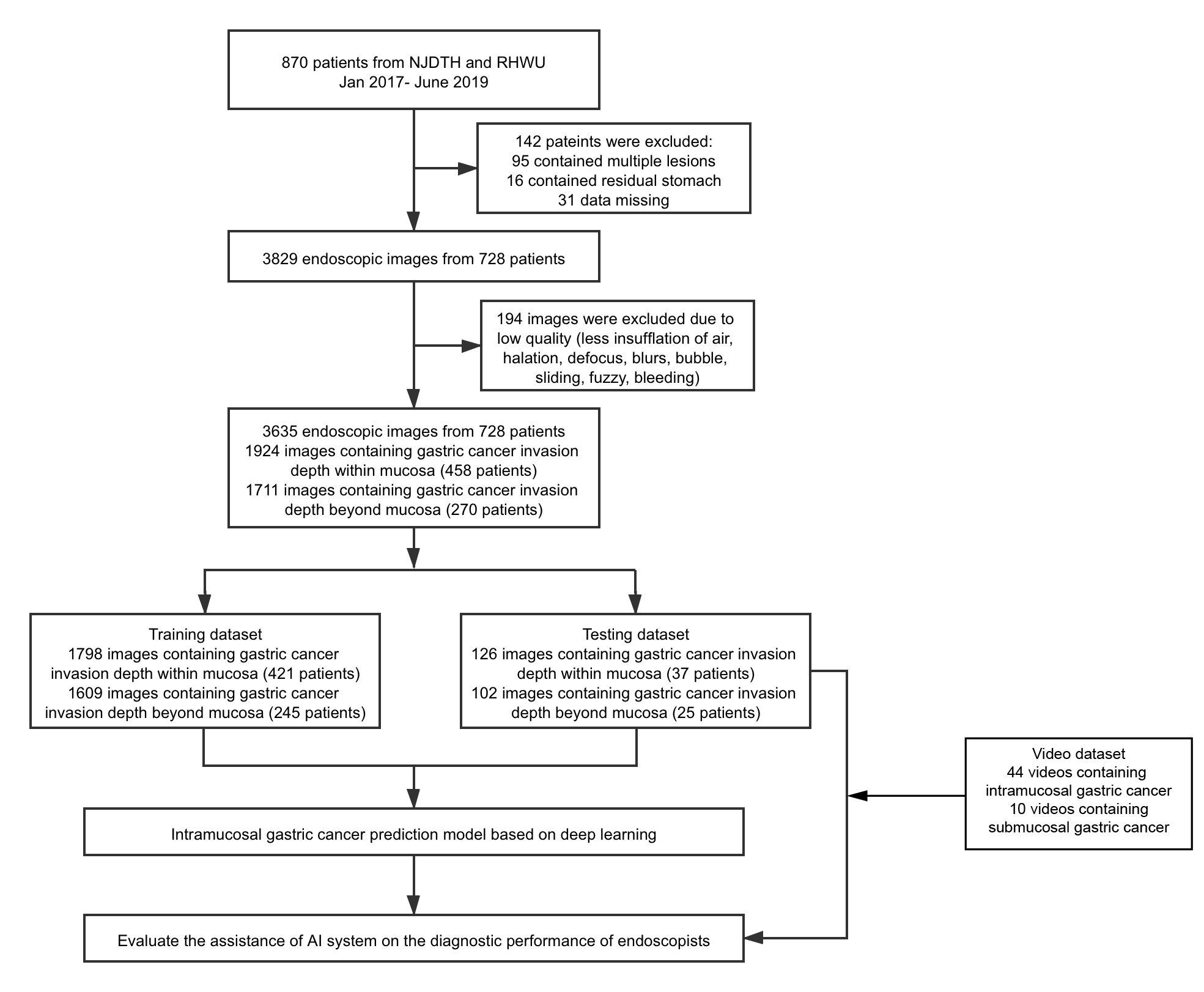

Supplement: Supplementary file 2 [file Image_1.tif]

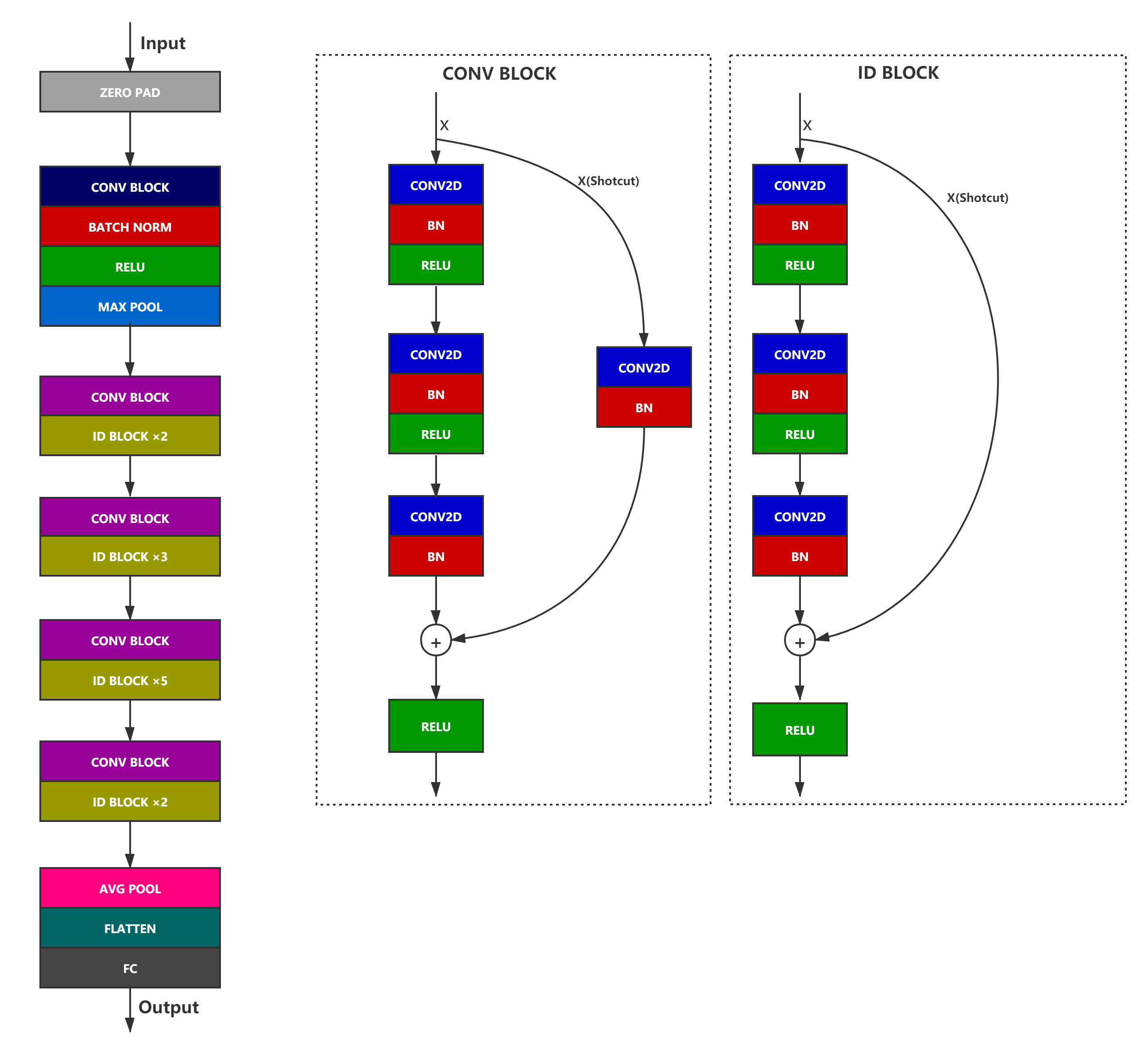

Supplement: Supplementary file 3 [file Image_2.tif]

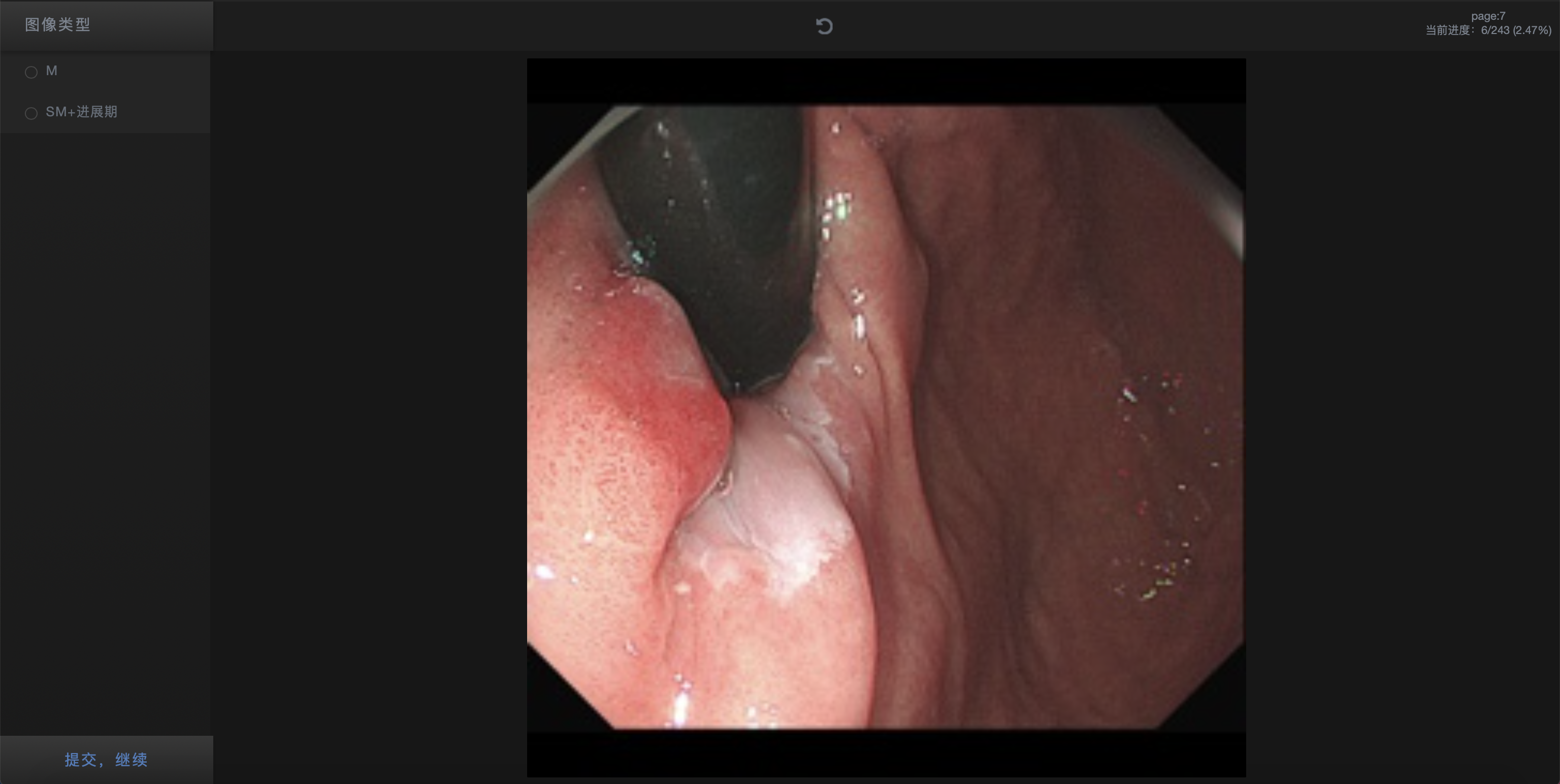

Supplement: Supplementary file 4 [file Image_3.tif]

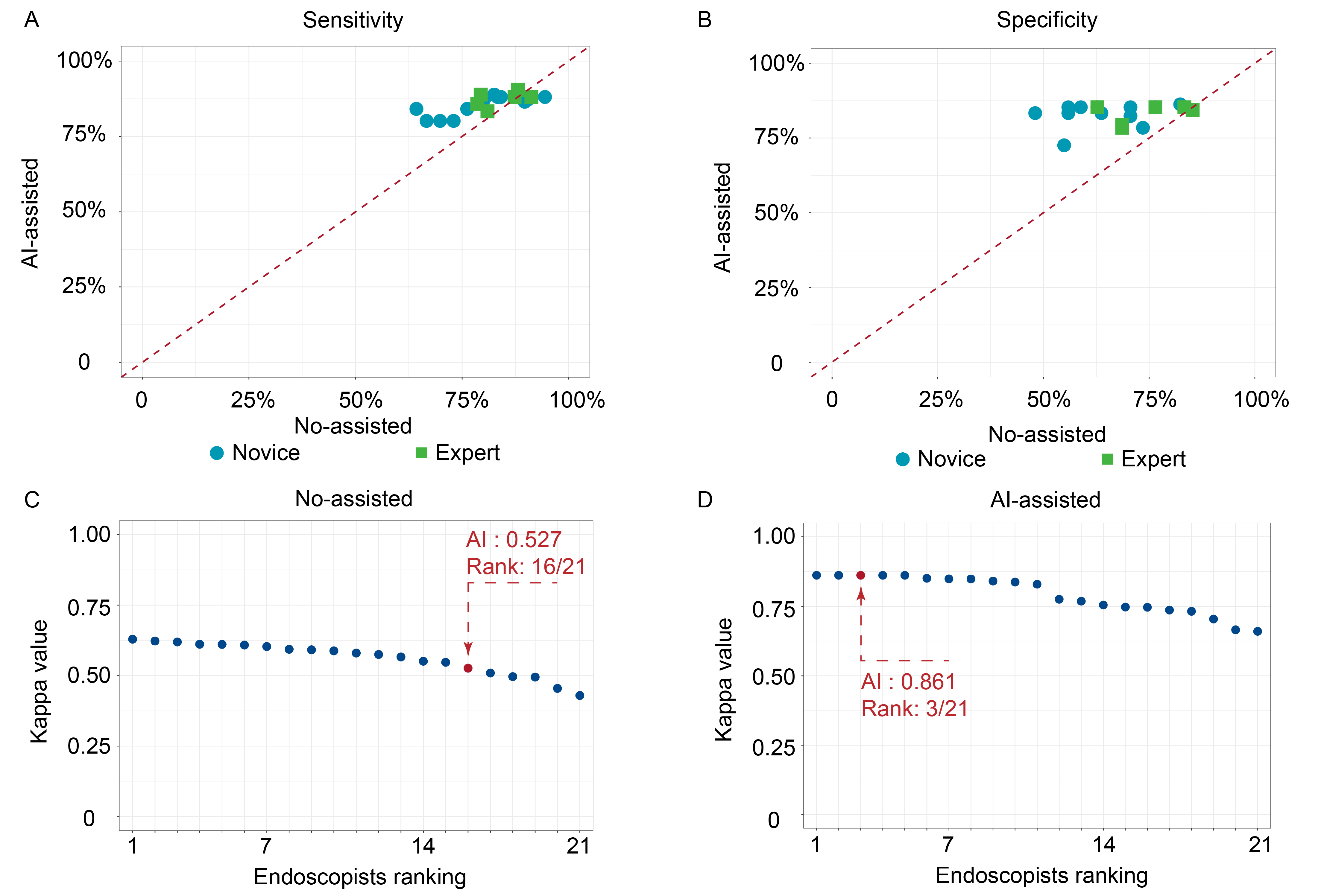

Supplement: Supplementary file 5 [file Image_4.tif]

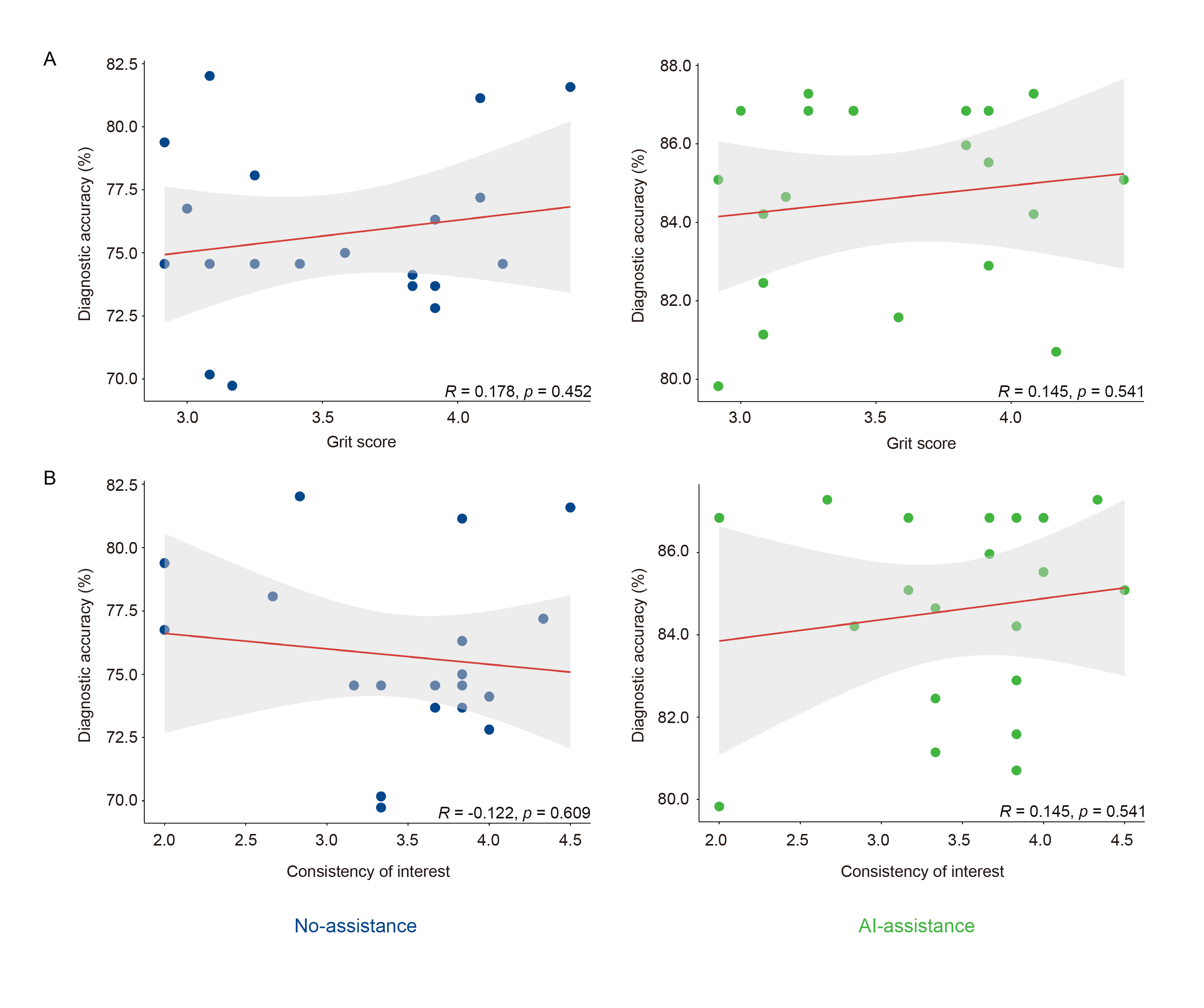

Supplement: Supplementary file 6 [file Image_5.tif]
